# Bridging epigenetics and pharmacology through systematic reviews tailored to WBS methodology: the triangle decision-making model as a pioneering translational biological drug delivery system

**DOI:** 10.3389/fmed.2025.1552904

**Published:** 2025-05-20

**Authors:** João Francisco Pollo Gaspary, Luis Felipe Dias Lopes, Antonio Geraldo Camara

**Affiliations:** ^1^Institute AuBento - Center for Education, Clinical Practice, and Research in Orthomolecular and Integrative Medicine, Santa Maria, Brazil; ^2^Center for Social and Human Sciences, Postgraduate Program in Administration, Federal University of Santa Maria, Santa Maria, Brazil; ^3^Institute Camara - Center for Clinical and Orthomolecular Practice, Ribeirão Preto, Brazil

**Keywords:** biological drug delivery system, translational research, personalized medicine, epigenetics, nutraceuticals, physical stimuli, medication adherence, inflammation reduction

## Abstract

**Background:**

The health industry plays a crucial role in improving the quality of life for individuals, continuously driving innovations in health service delivery. Translational research fosters intimate collaboration between scientists and medical professionals. A major obstacle to effective evidence-based treatments is drug adherence, prompting the search for innovative procedures to enhance drug delivery methods.

**Objectives:**

This study aimed to assess the impact of innovative drug delivery systems (DDS) based on physical stimuli on medication adherence among patients undergoing long-term treatments. The ultimate goal was to establish a framework-based approach to assist in clinical decision-making, enhancing drug absorption efficiency.

**Methods:**

Two systematic literature reviews (SLRs) was conducted across multiple databases, including PubMed, Scopus, and Web of Science, focusing on DDS activated by physical and biological stimuli. The research process was structured through the Work Breakdown Structure (WBS) methodology, dividing it into five interconnected Work Packages (WPs). Each WP explored specific aspects of the relationship between DDS and the human body.

**Results:**

The synthesis led to the development of the Triangle Decision-Making Model, a theoretical framework that prioritizes physiological balance to optimize drug delivery. The study underscores the importance of reducing insulin and cortisol levels to minimize inflammation and glycation, promoting an ideal state for drug absorption. The findings highlight the significance of using physical stimuli, such as hyperthermia, ultrasound-triggered drug delivery, and photodynamic therapy, to enhance drug bioavailability and target specificity.

**Conclusions:**

This research proposes a novel therapeutic intervention, grounded in systematic reviews and focused on improving drug delivery via physical stimuli. Using an open innovation approach, the triangular clinical decision model integrates personalized medicine and nutraceuticals, addressing epigenetics and nutrition's role in medication response. This framework aims to enhance drug absorption, metabolism, and targeted therapies, advancing treatment outcomes. Future studies should refine this model to promote homeostasis and validate its effectiveness across healthcare settings.

## 1 Introduction

The healthcare industry plays a pivotal role in advancing public health and improving overall quality of life (1). In recent years, the intricate relationship between epigenetics and pharmacology has garnered increasing attention, particularly in the context of chronic diseases and cancer (2–6). This dynamic interplay fosters innovation in healthcare services (1), often realized through a “from bench to bedside and back” approach. This translational process bridges the gap between scientific discovery and clinical application, requiring heightened interdisciplinary awareness and collaboration (7, 8). Its success hinges on the ability of basic scientists and clinical specialists to work together in an environment of mutual understanding and respect, ultimately advancing patient-centered treatments that integrate expert care with medical innovation (9–11).

As healthcare challenges become more complex and patient expectations evolve, the need for continuous innovation grows. One pressing issue in this landscape is medication non-adherence, extensively analyzed by Kardas et al. (12). Non-adherence significantly compromises therapeutic effectiveness, underscoring the urgency of developing innovative strategies to improve medication administration and integration into patients' lives. Kelly and Young (13) emphasized that successful innovation must be both usable and desirable. Given the established influence of epigenetic modifications on gene expression and drug response, integrating these insights into therapeutic strategies presents an opportunity to enhance treatment outcomes (2–6).

Reflecting the concerns raised by Kardas et al. (12), medication adherence remains critical for optimizing evidence-based therapies. Despite over five decades of extensive research and more than 130,000 scientific publications on non-adherence, a definitive solution has yet to be established (12). In this context, drug delivery systems (DDS) have been designed to transport therapeutic agents to their target sites within the body in a controlled and effective manner, improving both efficacy and adherence (14). These systems enhance treatment efficiency, minimize adverse effects, and optimize drug bioavailability, distribution, and release, ensuring maximum therapeutic benefit while mitigating risks associated with conventional administration (14–17). Such advancements hold significant potential for improving adherence to prescribed therapies.

Building on this foundation, the present study evaluates the impact of innovative DDS based on physical stimuli in enhancing medication adherence among patients undergoing long-term treatments. Rather than focusing on the discovery of new drugs, this research aims to optimize the administration of existing medications by improving their delivery to target cells, thereby maximizing therapeutic efficacy. The study explores the potential of physical stimuli to enhance drug bioavailability and effectiveness, linking these mechanisms to physiological balance and optimized cellular transport.

This research aims to determine how physical and/or physiological stimuli enhance pharmacological efficiency by optimizing membrane permeability and systemic biodisponibility, functioning as a biological drug delivery system. Addressing a critical gap in the literature, this study consolidates and analyzes evidence on drug delivery methods that optimize pharmacokinetics, particularly focusing on the role of emerging biological delivery systems. Ultimately, the research seeks to develop innovative solutions through design thinking (18) and open innovation methodologies (19), proposing a structured framework to support clinical decision-making. By refining drug absorption mechanisms, this approach aims to enhance therapeutic efficacy and provide a new perspective on optimizing pharmacological interventions in clinical practice.

## 2 Methods

**Figure 1:**
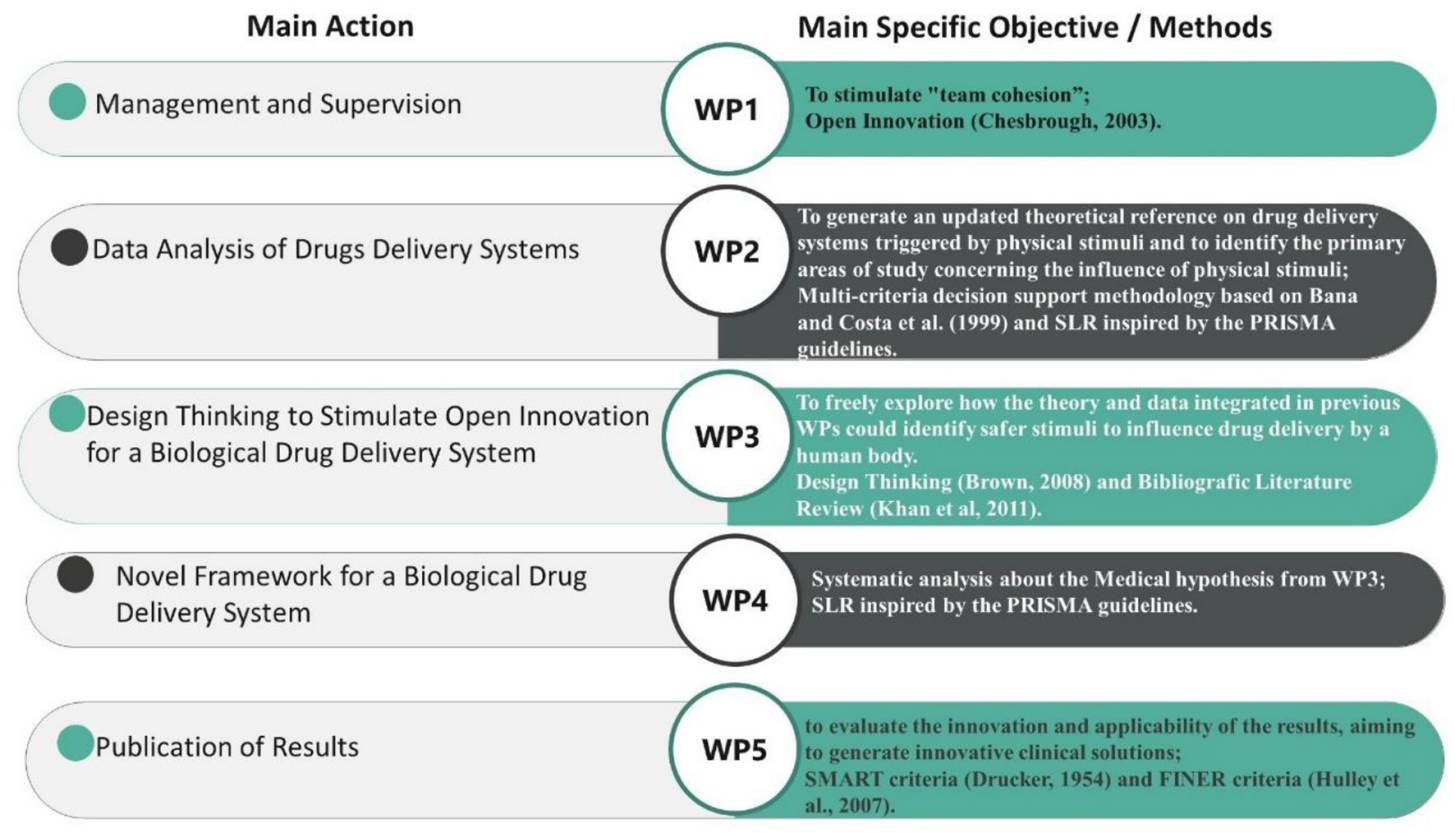
WBS methodology applied in this research.

To achieve the proposed objectives, this study adopted a Translational Research approach, structured through the Work Breakdown Structure (WBS) methodology, as described by the Project Management Institute (PMI) (2019; 2021) (20, 21). This management framework divided the research process into five interconnected Work Packages (WPs), each exploring specific aspects of the relationship between Drug Delivery Systems (DDS) and human physiology. The goal was to enhance drug absorption efficiency, ensuring a comprehensive and multidisciplinary perspective (Figure 1). Each WP was followed by a detailed qualitative analysis of the results to ensure their relevance and applicability in clinical contexts. The WBS methodology was chosen for its ability to add value to the innovation process, structuring the research step-by-step while maintaining a high scientific standard. This aligns with the principles discussed by Gaspary et al. (22), which emphasize the importance of tailoring research methodologies to specific organizational and scientific contexts to maximize their potential.

**Figure 2:**
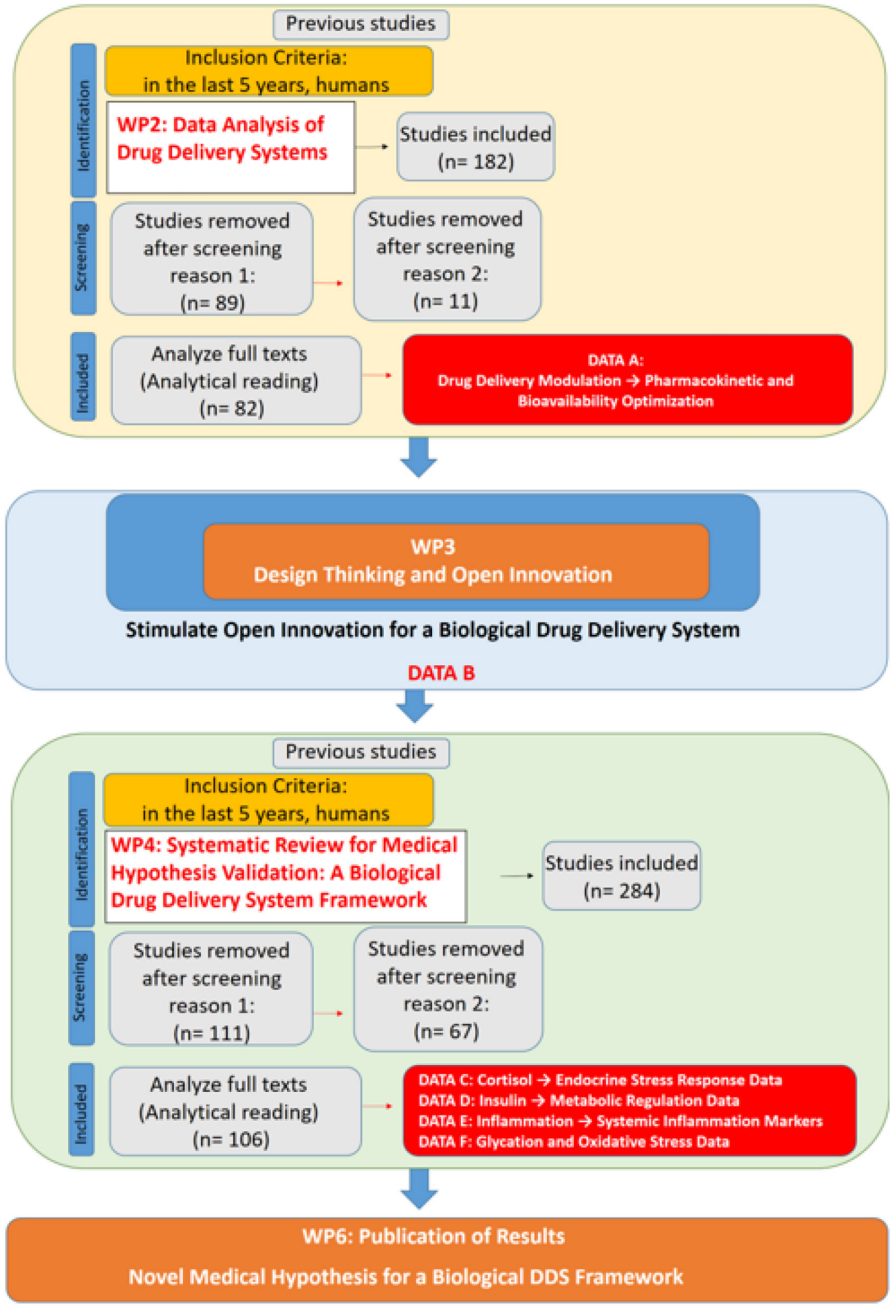
PRISMA-compliant flowchart integrating work breakdown structure (WBS) methodology and systematic reviews for the development of a novel medical hypothesis.

**WP1 (Management and Supervision)** ensure adherence to the initial objectives and timeline. It aimed to foster team cohesion through Open Innovation (19), continuously refining the WBS as the study progressed while maintaining clear feedback and communication mechanisms across all work packages. Additionally, WP1 assessed the impact of newly developed medical hypotheses. The methodological flow of this study, integrating the WBS framework with systematic reviews, is formally structured in Figure 2, which presents a PRISMA-compliant flowchart detailing the inclusion and exclusion of studies at each stage, ensuring transparency and reproducibility in the research process. Additionally, a specific flowchart for each stage (WP2 and WP4) will be presented separately to provide a detailed breakdown of the screening and selection process in each phase.

**Figure 3:**
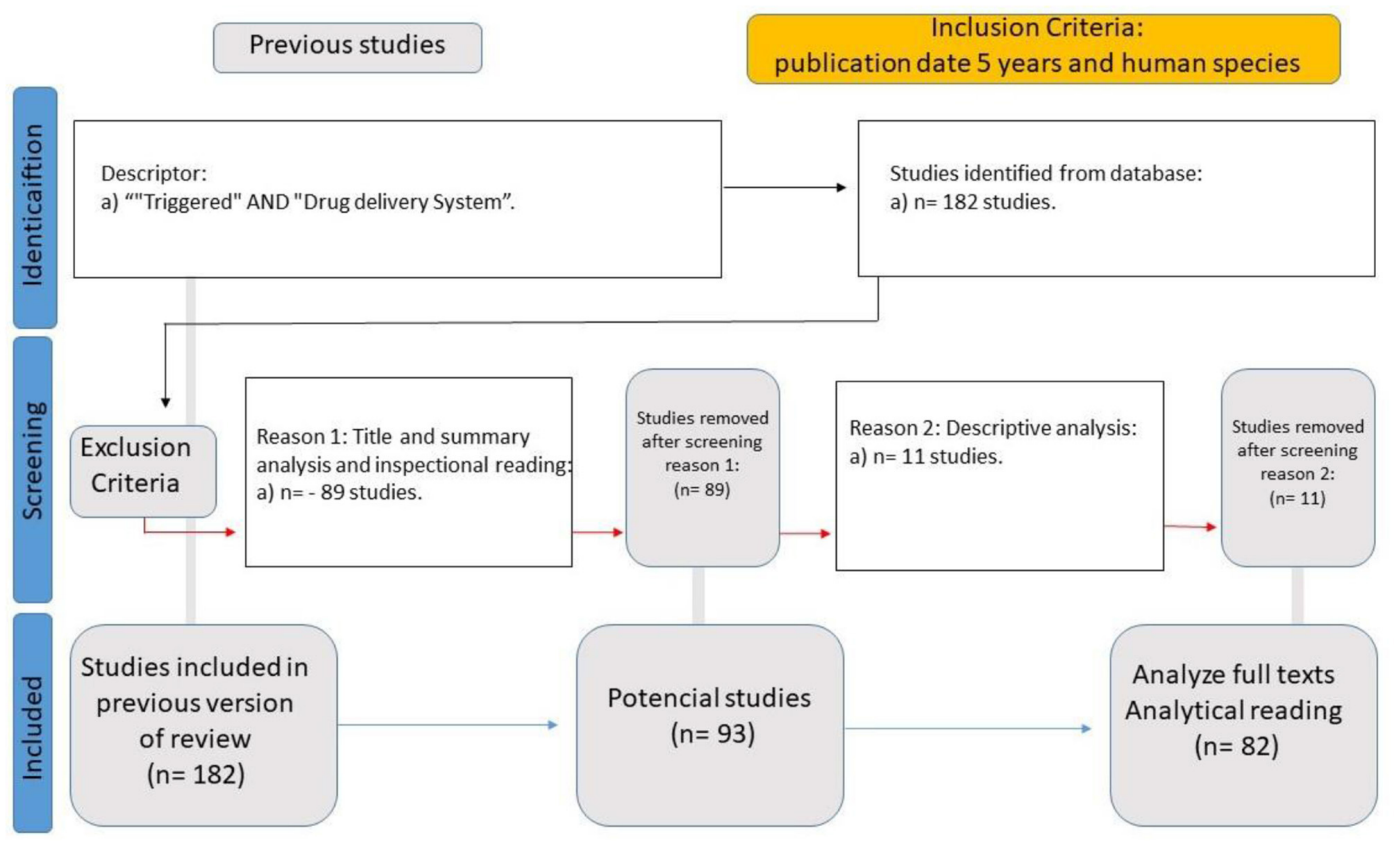
WP2 SLR flowchart.

**WP2 (Data Analysis of Drug Delivery Systems)** sought to identify the primary areas in which physical stimuli influence DDS by employing a multi-criteria decision support methodology based on Bana and Costa et al. (23). A systematic literature review (SLR) was conducted to gather data, applying the eligibility criteria of studies published within the last five years, focused on human subjects, and indexed under the descriptors “Triggered” AND “Drug Delivery System” in English. This search yielded 182 studies, of which 82 were selected for full analysis (Figure 3). The focus of data collection in this stage of the research was to identify parameters that enhance drug performance and systemic bioavailability. This analysis provided the foundation for WP3, which further explored the role of epigenetic changes as a potential enhancer or modulator, influencing biological responses to pharmacological interventions. In this context, epigenetic modifications are not merely a consequence of DDS application but may act as a facilitating mechanism for optimizing drug delivery and systemic distribution.

**WP3 (Design Thinking to Stimulate Open Innovation for a Biological Drug Delivery System)** explored how theoretical and empirical data integrated in previous work packages could inform the development of a biological DDS model based on physical stimuli. This phase applied Design Thinking (18) and Open Innovation (19) to establish connections between emerging theoretical insights and practical applications, emphasizing a human-centered approach to problem-solving. Design Thinking is an iterative process that involves empathy, problem definition, ideation, prototyping, and testing, ensuring continuous refinement based on user feedback and experimental validation (24). Open Innovation, in turn, promotes collaboration beyond conventional research environments, facilitating the exchange of ideas, resources, and technologies among researchers, clinicians, and other stakeholders (25). To support the development of this model, an additional literature review was conducted following the criteria proposed by Khan et al. (26). The selection criteria applied in this phase are detailed in Table 1.

**Table 1:** Article selection criteria in a review for this study based on Khan et al. (26) for WP3.

**Criteria**	**Variables**
Database	LILACS; Medline; Web of Science; Scopus; SciELO; Google Scholar; Research Gate; ClinicalTrials.gov; Patentscope; Prospero
Timeframe	All studies published until 2024
Languages	English, Portuguese, and Spanish
Indexed terms	Descriptors in English were generated from the iterative process.
Inclusion criteria for analysis	Broad, with the aim of idea testing

Design Thinking integrates user needs, technological capabilities, and business constraints, structuring problem-solving through inspiration, ideation, and implementation stages (24, 27–29). The iterative and collaborative nature of this methodology fosters experimentation and continuous refinement, particularly in developing DDS strategies (30–32). Its success in enhancing patient-centered healthcare solutions is well-documented across various clinical applications (29, 33, 34), reinforcing its applicability in designing therapeutic innovations. Open Innovation complements this approach by fostering external collaboration, enhancing knowledge absorption, and broadening the scope of problem-solving strategies (19, 25).

**Figure 4:**
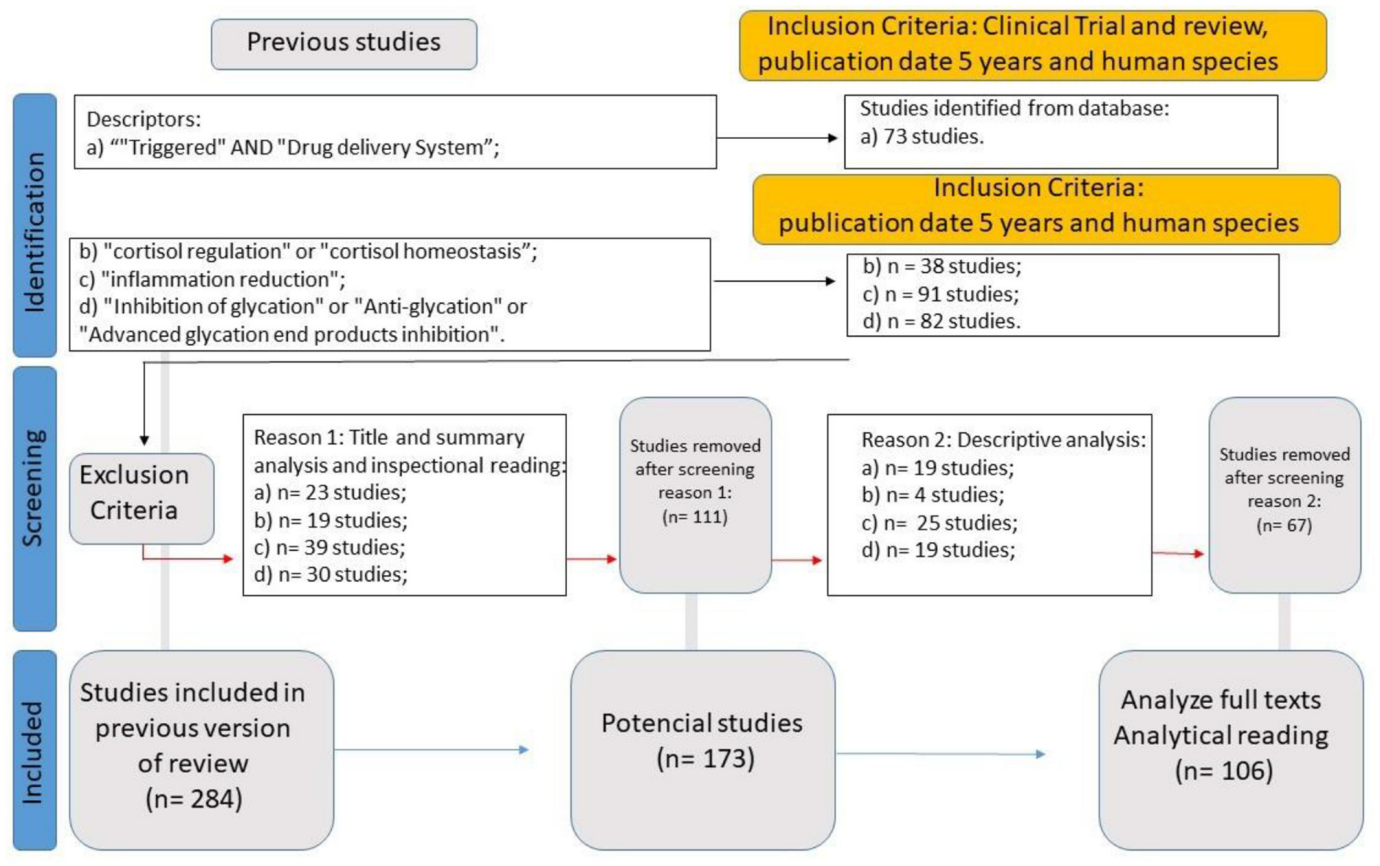
WP4 SLR flowchart.

**WP4 (Systematic Review for Medical Hypothesis Validation: A Biological Drug Delivery System Framework)** aimed to systematically evaluate the primary physiological mechanisms underlying drug absorption, assessing whether the framework developed in WP3 could be supported or refined based on existing evidence. A second systematic literature review was conducted to gather data using the following eligibility criteria: Clinical Trials or Review studies published within the last five years, focused on human subjects, indexed under the descriptors “insulin regulation” or “insulin homeostasis” (yielding 73 articles); “cortisol regulation” or “cortisol homeostasis” (38 articles); “inflammation reduction” (91 articles); and “inhibition of glycation” or “anti-glycation” or “advanced glycation end products inhibition” (82 articles). This process initially identified 284 studies, from which 106 were selected for full analysis (Figure 4).

The methodology employed in WP2 and WP4 was inspired by PRISMA guidelines (35) but tailored to align with the WBS framework, ensuring a structured and systematic approach to literature review and data synthesis. While the reviews were not formally registered, a detailed protocol was followed, outlining the search strategy, inclusion and exclusion criteria, data extraction procedures, and analysis methods. Table 2 summarizes these steps. Future research will consider formal registration to enhance the transparency and replicability of the review process.

**Table 2:** SLR methodology inspired by PRISMA guidelines and tailored to the WBS framework.

**PRISMA step**	**Description**
Sources and search strategy	Comprehensive search conducted in NIH (80), Scopus (81), and Web of Science (82) to capture key points.
Study selection	Two reviewers independently screened titles and abstracts for eligibility.
Data collection process	Standardized data extraction form used. Data included study characteristics, methodology, and key findings. The review focused on WP2 objectives: “triggered drug delivery systems” activated by stimuli (e.g., pH, temperature, enzymes, light). These systems are relevant to the Brazilian health system, enhancing treatment effectiveness and control. The initial examination included titles and abstracts, and the process was repeated to achieve the WP4 objectives.
Data items	Included definitions, types of biological impacts, and methodologies for measuring these influences for each WP's specific objectives.
Risk of bias in individual studies	Assessed methodological quality, measurement rigor, and evidence strength linking WP2 and WP4 objectives.
Summary measures and synthesis of results	Synthesized results narratively, highlighting biological influences, their measurement, and impact.
Additional analyses	Meta-analysis was not feasible due to study heterogeneity. A multi-criteria methodology was employed to analyze treatment network geometry and potential biases.
Exploration of treatment network geometry	Used multi-criteria methodology to visually and analytically represent the evidence network through tables and figures.
Identification and mitigation of potential biases	The multi-criteria methodology identified and addressed potential biases, facilitating objective data treatment. Noted limitations include broad inclusion criteria, language bias, and absence of a registered review protocol. These were mitigated through quality assessment and consensus discussions.
Exclusion criteria (WP2—drug delivery systems SLR)	Studies were excluded if they did not analyze the role of physical stimuli in DDS, lacked experimental or clinical validation, or focused exclusively on chemical-based delivery mechanisms without correlation to physical stimuli. Reviews without explicit methodological rigor or that did not present clear inclusion criteria were also excluded.
Exclusion criteria (WP4—clinical decision model SLR)	Excluded studies were those that lacked relevance to insulin and cortisol regulation, inflammation reduction, or glycation inhibition in a translational context. Studies focusing solely on genetic predisposition, without direct intervention or modulation of physiological responses, were also excluded. Additionally, research without clear quantitative or qualitative assessments of intervention effects was removed.
Absence of review registration and considerations for future research	While the systematic reviews in WP2 and WP4 were conducted following PRISMA principles, they were not formally registered in databases such as PROSPERO. This may limit visibility in repositories dedicated to systematic reviews. However, methodological rigor was maintained through structured eligibility criteria, independent reviewer assessment, and adherence to a multi-criteria framework. Future research will consider formal registration to further strengthen transparency and traceability.
Evidence base compilation and description	Presented FPVs and CSFs clearly and informatively using a multi-criteria methodology. Ensured a comprehensive description of the evidence base and its contribution to overall analysis.

To ensure methodological rigor, this study was structured according to systematic review principles, incorporating the PICO(S) framework for study design and data synthesis. The population targeted includes individuals with metabolic and inflammatory conditions potentially benefiting from biological drug delivery strategies. The intervention analyzed focuses on systemic physiological optimization to enhance drug bioavailability, particularly through cortisol and insulin regulation, inflammation reduction, and glycation inhibition. The comparison element derives from standard DDS approaches, which prioritize formulation-based mechanisms rather than physiological modulation. The outcomes were assessed through measurable biological markers associated with enhanced pharmacological efficiency. The study designs considered included systematic literature reviews and translational analyses of physiological mechanisms relevant to DDS effectiveness.

In addition to systematic literature analysis, a multi-criteria methodology (23, 36, 37) was applied to rigorously assess study quality. This involved an in-depth examination of study design, methodological rigor, and reported outcomes to identify potential biases or limitations, ensuring that the findings were grounded in high-quality evidence. The multi-criteria decision-making model of Bana e Costa et al. (23), as cited by Gerhardt et al. (37), provided the foundational framework for structuring the review. The information was categorized into three levels: Fundamental Point of View (FPV), Critical Success Factors (CSF), and Key Performance Indicators (KPI) (37).

The FPVs represented the strategic objectives for evaluating physical and biological stimuli in DDS, while CSFs identified essential areas influencing treatment success. KPIs were employed to quantify the effectiveness of different therapeutic interventions. This structured approach allowed for a balanced assessment of evidence, facilitating the development of a robust framework for evaluating DDS innovations (23, 37). The critical role of FPVs in decision-making, as outlined by Negreiros et al. (38), ensured that key strategic priorities were incorporated into the evaluation process. CSFs, following the principles established by Wong and Aspinwall (39), provided a foundation for assessing the practical implementation of DDS strategies. The integration of these elements facilitated a comprehensive evaluation of the treatment network's geometry and its potential applications in clinical practice.

While no meta-analysis was conducted due to heterogeneity in study designs, interventions, and outcome measures, the systematic categorization of findings provided a structured synthesis of existing knowledge. This approach ensured a nuanced understanding of the interplay between DDS strategies and physiological mechanisms, directly supporting the objectives of WP2 and WP4.

**WP5 (Publication of Results)** focused on evaluating the innovation and applicability of findings using SMART (40) and FINER (41) criteria to assess their potential for clinical implementation. This phase also addressed the methodological limitations and ethical considerations associated with the study, particularly regarding the use of patient data, to ensure transparency and adherence to scientific and ethical standards. By integrating multi-criteria decision-making, systematic literature analysis, and innovative research methodologies, this study sought to establish a framework that enhances the efficacy and adherence of drug delivery systems, providing a structured approach to advancing clinical practice.

## 3 Results

All results were obtained under the direct monitoring and integration of WP1 actions. As part of WP2, the first SLR included a comprehensive examination of 82 studies, which were assessed for methodological quality and risk of bias. Findings were synthesized narratively due to the heterogeneity of the study designs and outcomes reported. This narrative synthesis highlighted the diversity of physical and biological stimuli used in DDS and their effects on drug bioavailability and efficacy, providing a comprehensive overview of the current state of research in this field.

At this stage of the theoretical framework, the primary objective was to identify the main physical stimuli associated with DDS. Table 3 presents the categorized DDS, selected as Fundamental Points of View (FPVs) to align with this objective. The primary selection criterion was that the system should involve “physical stimulation” applicable in a real-world medical setting, ensuring its translational potential.

**Table 3:** Categorization of DDS Involving Physical Stimuli.

**FPV**	**Definition**	**Recent studies**
Hyperthermia	A therapeutic approach involving the controlled elevation of temperature in specific body regions to enhance drug delivery, improve tissue permeability, and increase the effectiveness of certain treatments, particularly in oncology.	(83–85)
Ultrasound-triggered drug delivery	A technique that utilizes ultrasonic waves to enhance drug penetration and control release at targeted locations. When exposed to ultrasound, drug-loaded nanoparticles or microbubbles undergo mechanical vibrations, facilitating localized and efficient drug activation.	(86, 87)
Photodynamic therapy	A treatment modality combining a photosensitizing agent with light exposure at a specific wavelength to induce therapeutic effects. This approach is commonly used in oncology and dermatology, where light activation leads to reactive oxygen species (ROS) production, selectively destroying targeted cells.	(88, 89)
pH-triggered drug delivery	A system in which drug release is regulated by environmental pH variations. This approach takes advantage of the natural pH differences in various tissues and biological compartments to achieve targeted drug release, enhancing therapeutic precision while minimizing systemic side effects.	(90, 91)
Magnetically triggered drug delivery	A delivery system where drug release or activation is modulated by external magnetic fields. This method employs magnetically responsive nanoparticles or carrier systems that are guided or activated by applied magnetic forces, improving site-specific drug accumulation and therapeutic efficacy.	(83, 92, 93)

FPV, Fundamental Point of View.

Additionally, as part of WP2, Table 4 outlines the parameters deemed critical for the therapeutic success of a drug delivery system. These parameters, classified as Critical Success Factors (CSFs), were systematically analyzed to identify the physiological conditions and mechanisms that directly influence DDS performance.

**Table 4:** Critical success factors as physiological parameters for therapeutic success in DDS.

**CSF**	**Definition**	**Recent studies**
Complex cellular pathways	These pathways govern drug absorption, distribution, metabolism, and excretion, directly influencing therapeutic efficacy. Understanding these mechanisms allows the development of more precise and efficient DDS strategies.	(94, 95)
Cell homeostasis	Maintaining intracellular equilibrium is essential to optimizing drug effectiveness while minimizing side effects. Disruptions in homeostasis can impair drug transport and metabolism.	(96, 97)
Oxidative balance	The redox state of cells modulates drug activation and detoxification. Controlling oxidative stress enhances drug efficacy while preventing cellular damage.	(96, 98)
pH Regulation	The pH gradient across tissues and organelles affects drug solubility, stability, and targeted release. Designing pH-responsive DDS improves site-specific drug activation.	(90, 99)
Electromagnetic effects	External electromagnetic fields can regulate drug transport across membranes, facilitating precise, localized delivery. This strategy enhances drug targeting and minimizes systemic exposure.	(93, 100)
Physical stimuli	Light, heat, magnetic fields, and ultrasound are key physical triggers for controlled drug release, offering enhanced spatial and temporal precision in therapy.	(101, 102)
Intracellular transport	Optimizing intracellular pathways ensures drugs reach their intended targets within cells, improving bioavailability and therapeutic outcomes.	(103, 104)
Physiological cascade	Sequential biochemical events amplify drug responses, creating synergistic therapeutic effects and optimizing DDS efficiency.	(102, 105)
Precision medicine	Tailoring drug delivery based on individual genetic, metabolic, and physiological profiles ensures higher treatment efficacy and reduced adverse effects.	(100, 106)
Immune response	Understanding the interaction between the immune system and DDS allows for immunomodulatory strategies that enhance drug effectiveness and minimize adverse immune reactions.	(107, 108)
Cellular uptake pathway	Optimizing drug penetration across biological barriers enhances site-specific accumulation and therapeutic efficacy while reducing systemic toxicity.	(109)
Mitochondrial stimulation	Targeting mitochondria in DDS enables direct intervention in metabolic and degenerative disorders, improving therapeutic precision and cellular energy balance.	(95, 110)
Enhancement of zeta potential	Modulating zeta potential improves drug stability, bioavailability, and interaction with target cells, optimizing drug dispersion and cellular uptake.	(111)
Antioxidant agents	Incorporating antioxidants into DDS protects drugs and tissues from oxidative damage, stabilizing formulations and enhancing therapeutic benefits.	(112)
Tunable photoactivity	Light-activated drug release offers precise spatiotemporal control, reducing systemic exposure and enabling personalized therapeutic approaches.	(113)

CSF: Critical Success Factors.

The WP2 protocol also identified a set of therapeutic application domains to illustrate the primary diseases and clinical conditions in which DDS employing physical stimuli have shown promising results. Rather than functioning as traditional Key Performance Indicators (KPIs), these domains serve as evidence-based use cases, guiding the translational applicability of the model. These clinical contexts are summarized in Table 5.

**Table 5:** Clinical application domains of DDS based on physical stimuli.

**Therapeutic application domain**	**Description**	**Recent studies**
Cancer	A multifactorial disease driven by genetic mutations, epigenetic alterations, and environmental factors, leading to uncontrolled cellular proliferation, invasion, and metastasis. Effective drug delivery systems are crucial for improving targeted therapy and minimizing systemic toxicity.	(114–116)
Infections	Diseases caused by pathogenic microorganisms, including bacteria, viruses, fungi, and parasites. Targeted DDS enhance antimicrobial efficacy, reduce resistance development, and improve patient compliance.	(117, 118)
Cardiovascular disease	A broad category encompassing conditions such as coronary artery disease, heart failure, hypertension, and stroke. Drug delivery strategies can optimize therapeutic agents targeting vascular remodeling, inflammation, and oxidative stress.	(119–121)
Chronic inflammatory diseases	Persistent inflammatory conditions, such as rheumatoid arthritis, inflammatory bowel disease, and psoriasis, driven by immune dysregulation. Controlled DDS can improve drug targeting to inflamed tissues, reducing systemic adverse effects.	(122, 123)
Biomedical applications	The application of biotechnological and medical innovations, such as tissue engineering, regenerative medicine, and advanced diagnostics, to enhance therapeutic precision and efficiency.	(88, 124, 125)
Drug resistance	The ability of cancer cells or microorganisms to evade therapeutic effects through genetic and adaptive mechanisms. DDS can enhance drug bioavailability, target resistant populations, and delay resistance onset.	(94, 126, 127)
Synergistic therapy	The concurrent application of multiple therapeutic strategies (e.g., chemotherapy and immunotherapy) to enhance efficacy, reduce resistance, and improve clinical outcomes. DDS facilitate controlled release and co-administration of complementary agents.	(128–130)
Immunotherapy	A therapeutic approach leveraging the immune system to recognize and eliminate malignant or infected cells, including immune checkpoint inhibitors, monoclonal antibodies, and adoptive cell therapies. DDS improve targeting, reducing off-target effects.	(108, 131)
Mitochondrial dysfunction	Deficiencies in mitochondrial bioenergetics and homeostasis implicated in neurodegenerative, metabolic, and inflammatory disorders. Targeted DDS can modulate mitochondrial pathways to restore cellular function.	(95, 110, 132)
Oxidative stress	A pathological state resulting from excess reactive oxygen species (ROS) and insufficient antioxidant defenses, contributing to aging, cancer, and cardiovascular diseases. Antioxidant-loaded DDS can mitigate oxidative damage and enhance therapeutic outcomes.	(95, 122)

The integration of WP2 findings provided the foundation for WP3, which applied Design Thinking to the structured interrelation of FPVs, CSFs, and KPIs. The WP2 review supports the continued investigation of innovative DDS, despite limitations related to study heterogeneity. Notably, approaches focused on optimizing body pH and zeta potential through DDS demonstrated particular promise. Translational research on DDS facilitated the exploration of multiple hypotheses to enhance existing clinical therapies. The hypotheses selected for this study were based on identified physiological response patterns to specific stimuli, aligned with the research objectives.

WP3 specifically analyzed how each FPV and CSF from WP2 influenced acid-base balance, considering cellular, tissue, and systemic pH as reference parameters. Acid-base homeostasis is fundamental to physiological stability, cellular metabolism, and overall function. Dysregulation of plasma pH can lead to significant physiological disturbances, reinforcing the need for precise modulation of this parameter (42). This rationale led to the first formulated Medical Hypothesis (MH A): optimizing body pH is crucial for an effective drug delivery system.

Additionally, zeta potential emerged as a key determinant of therapeutic efficacy. Given that water constitutes ~50–70% of total body weight (42), the stability of colloidal dispersions—characterized by zeta potential—directly impacts molecular interactions in biological systems. The zeta potential quantifies the electrical charge near the interface of colloidal particles, influencing their stability, distribution, and biological interactions (43). While originally described in physicochemical systems, its role in biomedical research is increasingly recognized, particularly concerning nanoparticles and controlled drug release systems (44–46). Thus, in a translational reinterpretation, MH B was formulated: optimization of body zeta potential enhances the therapeutic effects of DDS. Table 6 summarizes the ideation process and initial considerations regarding the direction of future DDS research.

**Table 6:** Stages of WP3 design thinking and their correlation with this study.

**Idea generation**	**Main associated points of view**	**Applicable field of medical practice**
Optimizing body pH (MH A)	Hyperthermia Ultrasound-triggered drug delivery Photodynamic therapy pH-triggered drug delivery Magnetically triggered drug delivery	Orthomolecular (56–58) Integrative (133–135) Complementary (136)
Body zeta potential optimization (MH B)	pH-triggered drug delivery Magnetically triggered drug delivery	Orthomolecular (58)

MH, medical hypothesis.

**Figure 5:**
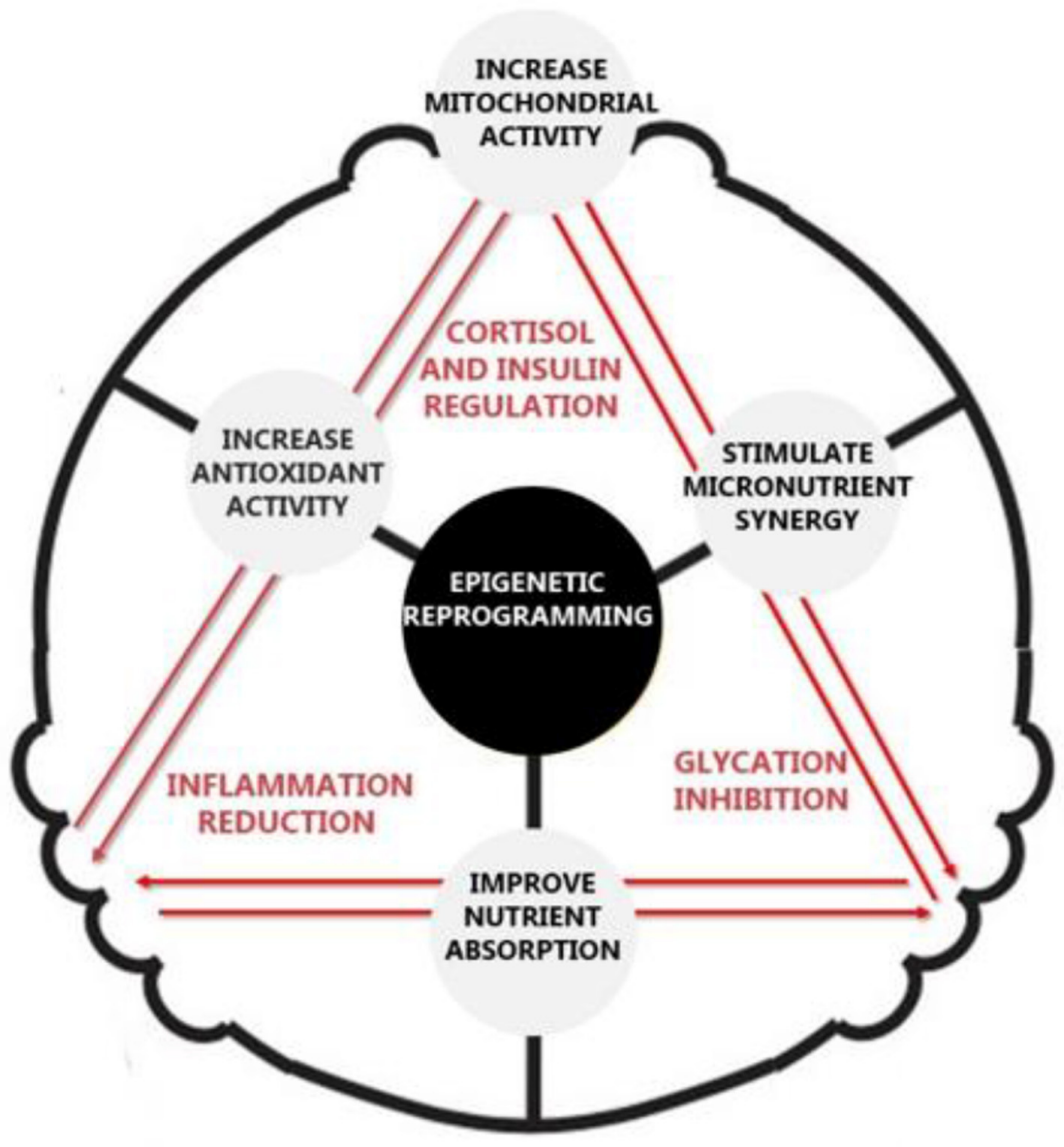
The triangular clinical decision-making model.

Following the Design Thinking process, the WP3 actions progressed with the implementation of the Open Innovation methodology, resulting in the construction of a theoretical model for clinical decision-making aimed at optimizing body pH and body potential. This novel approach establishes an optimized body balance by applying the triangular clinical decision model, functioning as a Biological DDS. This framework is structured around three main vertices: Cortisol and Insulin Regulation, Inflammation Reduction, and Glycation Inhibition, as illustrated in Figure 5. As part of the WP3 tasks, the framework underwent its first literature review, as outlined in Table 1, for an initial assessment of its viability. The initial conclusions, summarized in Table 6, motivated the commencement of WP4.

The initial conclusions motivating this phase are summarized in Table 7, providing a structured overview of the primary findings that guided the subsequent evaluation steps. WP4 was tasked with evaluating, through a systematic literature review (SLR), whether the model developed in WP3 could function as a viable and biologically integrated DDS. This phase involved reassessing WP2 CSFs from the perspective of the proposed system, systematically analyzing the individual contributions of each vertex in the decision-making model.

**Table 7:** Initial conclusions from the literature review motivating the commencement of WP4.

**Action**	**Evidence base**
Cortisol and insulin regulation	The interplay between cortisol and insulin is critical for metabolic homeostasis and overall physiological balance. Cortisol, a glucocorticoid hormone, modulates stress responses, while insulin regulates glucose metabolism. Dysregulation of either hormone contributes to metabolic disorders such as diabetes, obesity, and chronic stress-related pathologies. Studies confirm that interventions aimed at modulating these hormones can enhance metabolic control and improve therapeutic outcomes (137).
Inflammation reduction	Chronic inflammation is a key driver of numerous pathologies, including cardiovascular diseases, neurodegeneration, and cancer. Anti-inflammatory strategies, ranging from pharmacological interventions (e.g., NSAIDs, corticosteroids) to lifestyle modifications (e.g., dietary changes, physical activity, and physiotherapy), have been extensively documented. Controlling systemic inflammation is crucial for optimizing the internal environment for drug efficacy and cellular function (5, 138).
Glycation inhibition	The accumulation of advanced glycation end products (AGEs) accelerates aging and contributes to metabolic and neurodegenerative diseases. Nutritional and pharmacological interventions aimed at reducing glycation, such as dietary restriction of high-glycemic foods, supplementation with antiglycation agents (e.g., carnosine, pyridoxamine), and the use of AGE inhibitors, have been well-documented as potential strategies for mitigating glycation-related damage (139).
Optimization of body zeta potential	Zeta potential plays a crucial role in cellular interactions, nanoparticle stability, and drug delivery. Research suggests that optimizing the body's zeta potential can enhance membrane integrity, improve ion transport, and facilitate controlled drug release. This principle has been increasingly applied in nanomedicine, where electrostatic interactions influence drug stability, bioavailability, and targeted delivery (45, 46, 139–142).
Verisimilitude of proposed actions	The triangular clinical decision-making model is scientifically grounded, integrating evidence-based physiological mechanisms with innovative therapeutic applications. The proposed interventions align with existing data on biological drug delivery systems (DDS) that leverage physical and biochemical stimuli to enhance drug absorption and systemic regulation. The framework's validity is supported by established scientific literature, reinforcing its translational potential.
Integration and conclusion	The proposed triangular model introduces a novel paradigm for DDS, incorporating **body pH optimization, cortisol and insulin regulation, inflammation reduction, glycation inhibition, and zeta potential modulation**. By integrating these biological parameters, this approach has the potential to **redefine drug delivery methodologies**, bridging pharmacological therapy with personalized medicine and systemic health optimization.

The first WP4 analysis identified “Complex Cellular Pathways” and “Cell Homeostasis” as the most influential CSFs in insulin regulation. Numerous studies reinforced the central role of intricate cellular signaling networks in maintaining insulin homeostasis. For example, Lecorguillé et al. (47) demonstrated a strong link between glycemic control during pregnancy and DNA methylation patterns in neonatal cord blood, illustrating how cellular homeostasis impacts metabolic regulation. Similarly, Solinas and Becattini (48) highlighted the importance of dietary interventions in modulating insulin response, emphasizing that insulin regulation is contingent upon highly coordinated cellular mechanisms. These findings are summarized in Table 8.

**Table 8:** WP4 analysis 1: interrelationship between insulin regulation and WP2 CSFs.

**CSF**	**Description**	**Recent studies**
Complex cellular pathways	Insulin regulation relies on intricate signaling networks that control glucose uptake, insulin secretion, and cellular responsiveness. Disruptions in these pathways contribute to insulin resistance and metabolic dysregulation.	(48, 68, 79, 143–145)
Cell homeostasis	Maintaining a stable intracellular environment is fundamental for insulin signaling, glucose metabolism, and cellular energy balance. Dysregulated homeostasis contributes to metabolic disorders, including diabetes.	(48, 62, 146–149)
Oxidative balance	Oxidative stress impairs insulin sensitivity, exacerbating metabolic dysfunction. Maintaining redox balance through antioxidant mechanisms supports insulin action and glucose metabolism.	(48, 68, 143–145, 149–152)
Intracellular transport	Insulin and glucose transport mechanisms across cell membranes are critical for maintaining glycemic control and ensuring effective glucose uptake by tissues.	(62, 63)
Physiological cascade	The insulin response involves sequential physiological events triggered by metabolic and hormonal signals, ensuring efficient glucose regulation.	(145, 152, 153)
Precision medicine	Tailoring diabetes treatment based on genetic predisposition and patient-specific biomarkers enhances therapeutic outcomes and optimizes glycemic control.	(150, 154)
Immune response	Inflammatory cytokines interfere with insulin signaling pathways, contributing to insulin resistance. Modulating immune response is crucial for restoring insulin sensitivity and metabolic function.	(62, 63, 151, 155, 156)
Mitochondrial stimulation	Mitochondrial efficiency is vital for ATP production and metabolic regulation. Dysfunctional mitochondria impair insulin action, highlighting the importance of mitochondrial stimulation in metabolic health.	(47, 149)

The second WP4 analysis revealed “Physiological Cascade” and “Intracellular Transport” as pivotal CSF's in cortisol regulation. Studies frequently underscored the importance of intracellular transport mechanisms and regulatory cascades in modulating cortisol balance. For instance, Weiss et al. (49) examined the impact of antenatal corticosteroid exposure on neonatal cortisol regulation, emphasizing the role of intracellular pathways. King et al. (50) explored physiological cascades involved in cortisol production, shedding light on stress-response mechanisms. Bhatt et al. (51) investigated the relationship between PTSD and cortisol regulation, proposing targeted interventions in precision medicine. Table 9 presents a synthesis of these results.

**Table 9:** WP4 analysis 2: interrelationship between cortisol regulation and WP2 CSFs.

**CSF**	**Description**	**Recent studies**
Complex cellular pathways	Cortisol regulation is influenced by intricate hormonal interactions and neuroendocrine pathways, particularly in response to stress and maternal mental health. Disruptions in these pathways can lead to long-term metabolic and psychological consequences.	(51, 64, 157)
Cell homeostasis	Cortisol plays a fundamental role in maintaining homeostasis, modulating energy metabolism, immune responses, and neurological function. Chronic stress disrupts these mechanisms, leading to systemic dysregulation.	(50, 51)
Oxidative balance	Cortisol secretion is closely linked to oxidative stress. Prenatal exposure to tobacco and marijuana has been shown to disrupt oxidative balance, altering cortisol responses and increasing susceptibility to metabolic and neurodevelopmental disorders.	(158)
pH regulation	Dysregulation of the hypothalamic-pituitary-adrenal (HPA) axis, particularly in COVID-19 patients, has been associated with disturbances in systemic pH levels, indicating a link between cortisol homeostasis and acid-base balance.	(159)
Physical stimuli	Physical exercise is a potent modulator of cortisol levels, demonstrating how controlled physical stimuli can positively influence the stress response and restore endocrine balance.	(160, 161)
Intracellular transport	Cortisol transport across cellular membranes is crucial for its bioavailability and function. Stress and endocrine dysregulation have been shown to impact intracellular cortisol distribution, affecting tissue-specific responses.	(49, 64–67)
Physiological cascade	The cortisol response is modulated by a complex cascade of hormonal and metabolic signals. Dysfunctions in this cascade contribute to pathological conditions such as PTSD, metabolic syndrome, and neuropsychiatric disorders.	(49–51, 65, 162–164)
Precision medicine	Individualized approaches to cortisol regulation are essential for precision medicine, particularly in psychiatric and metabolic disorders. Genetic predisposition and epigenetic factors influence cortisol sensitivity and therapeutic response.	(162, 165, 166)
Immune response	Cortisol exerts immunomodulatory effects, influencing inflammation and immune tolerance. Studies highlight the interaction between maternal cortisol levels and infant stress regulation, demonstrating long-term implications for immune function.	(167)

In the context of inflammation reduction, “Immune Response” and “Cell Homeostasis” emerged as the most significant CSFs. These elements were consistently identified as critical factors in mitigating inflammation-associated pathologies. Sommer et al. (52) demonstrated that immune modulation effectively reduced inflammation in experimental models, reinforcing the direct relationship between immune balance and cellular homeostasis. Santos (53) examined the regenerative potential of stem cell-based therapies, further illustrating the necessity of maintaining cellular equilibrium in inflammatory responses. Table 10 details the core findings of this analysis.

**Table 10:** WP4 analysis 3: interrelationship between inflammation reduction and WP2 CSFs.

**CSF**	**Description**	**Recent studies**
Complex cellular pathways	Inflammation reduction modulates complex cellular pathways by downregulating pro-inflammatory cytokine production and signaling, preserving the integrity of intracellular communication and homeostasis.	(52, 69–73, 168, 169)
Cell homeostasis	Controlling inflammation supports cellular homeostasis by preventing metabolic stress, maintaining membrane stability, and ensuring optimal function of biochemical pathways necessary for cellular survival.	(53, 69–73, 168)
Oxidative balance	Inflammatory processes contribute to oxidative stress by increasing reactive oxygen species (ROS). Suppressing inflammation restores redox homeostasis, minimizing oxidative damage and enhancing antioxidant defense mechanisms.	(53, 70–74, 168)
Physiological cascade	Lowering inflammation stabilizes the systemic physiological cascade by reducing chronic stress, regulating hormone secretion, and preventing excessive immune activation, which impacts metabolic and cardiovascular health.	(70–76, 169, 170)
Physical stimuli	Anti-inflammatory interventions improve the body's response to physical stimuli by reducing pain, swelling, and tissue damage, thereby enhancing mobility and musculoskeletal function.	(53, 70–76, 168)
Intracellular transport	Inflammation alters intracellular transport by affecting vesicular trafficking and endocytic pathways. Modulating inflammatory mediators improves cellular uptake and drug distribution.	(52, 69–76)
Precision medicine	Anti-inflammatory therapies benefit from personalized medicine approaches, allowing tailored treatments based on genetic, metabolic, and immunological profiles to optimize patient outcomes.	(70–76, 169–172)
Immune response	Regulating inflammation prevents excessive immune activation, balancing pro- and anti-inflammatory pathways to mitigate autoimmune disorders and chronic inflammatory diseases.	(52, 69–76, 170–172)
Antioxidant agents	The inclusion of antioxidants in therapeutic strategies helps reduce inflammation by neutralizing free radicals, protecting cellular components, and mitigating damage associated with chronic oxidative stress.	(70–72, 168)
Tunable photoactivity	Light-based modulation of inflammatory processes allows precise targeting of affected tissues, minimizing collateral damage and improving therapeutic outcomes in conditions such as arthritis and dermatological diseases.	(73, 173)

Regarding glycation inhibition, multiple CSFs were identified as key contributors to mitigating its pathological effects. These included complex cellular pathways, oxidative balance, intracellular transport, immune response, mitochondrial stimulation, and antioxidant activity. Tang et al. (54) illustrated how advanced glycation end-products (AGEs) disrupt cellular function, while Cheng et al. (55) emphasized the importance of oxidative balance in reducing glycation-induced damage. Although factors such as pH regulation and electromagnetic effects appeared less directly influential, emerging evidence suggests that physical stimuli and tunable photoactivity hold potential for further exploration. Table 11 provides a detailed overview of these findings.

**Table 11:** WP4 analysis 4: interrelationship between glycation inhibition and WP2 CSFs.

**CSF**	**Description**	**Recent studies**
Complex cellular pathways	Inhibiting glycation prevents the accumulation of advanced glycation end-products (AGEs), preserving proper cell signaling and maintaining biochemical pathways essential for cellular function.	(54, 55, 174–178)
Cell homeostasis	By reducing glycation, cellular homeostasis is preserved, preventing metabolic dysregulation and protein misfolding that can lead to impaired function and cell death.	(54, 55, 179–184)
Oxidative balance	Glycation enhances oxidative stress by promoting reactive oxygen species (ROS) production. Its inhibition reduces oxidative damage, sustaining the antioxidant defense system.	(77, 78, 174, 185–188)
pH regulation	Glycation inhibition stabilizes cellular pH by preventing the accumulation of acidic by-products that contribute to metabolic acidosis and cellular dysfunction.	(189, 190)
Physical stimuli	Preventing glycation preserves tissue elasticity and biomechanical properties, maintaining structural integrity and responsiveness to therapeutic interventions.	(180, 190, 191)
Intracellular transport	Inhibiting glycation protects cytoskeletal and transport proteins, ensuring effective intracellular trafficking of biomolecules and drugs.	(54, 55, 174, 178, 179, 181)
Physiological cascade	By preserving enzyme activity and receptor function, glycation inhibition maintains regulatory physiological cascades essential for metabolic homeostasis.	(54, 175)
Precision medicine	The inhibition of glycation enhances personalized therapeutic strategies by targeting glycation-specific biomarkers, improving tailored interventions.	(192, 193)
Immune response	Glycation inhibition mitigates pro-inflammatory AGEs, reducing chronic immune activation and preserving immune system function.	(174, 190, 192, 194, 195)
Cellular uptake pathway	Preventing glycation preserves membrane receptor function, optimizing drug absorption and cellular nutrient uptake.	(54, 174, 178, 179, 181, 196)
Mitochondrial stimulation	Glycation-induced mitochondrial dysfunction is counteracted, preserving ATP production and cellular energy balance.	(177, 178, 181, 183)
Enhancement of zeta potential	Reducing glycation improves zeta potential, optimizing electrostatic interactions critical for cellular communication and homeostasis.	(54)
Antioxidant agents	The inhibition of glycation reduces oxidative burden, complementing antioxidant therapies that mitigate AGE-induced damage.	(54, 77, 78, 174, 181, 185–188)
Tunable photoactivity	By maintaining protein integrity, glycation inhibition enhances the precision of light-based therapies, improving their efficiency in diagnostics and treatment.	(180, 190)

Based on WP4's analysis of 106 reviewed studies, the integration of insulin and cortisol regulation, inflammation reduction, and glycation inhibition as a biological DDS is supported by extensive scientific literature. Modulating these pathways creates a more balanced internal environment, enhancing drug absorption and therapeutic efficacy. This approach aligns with the principles of personalized medicine, suggesting that systemic regulation can significantly improve clinical outcomes.

**Figure 6:**
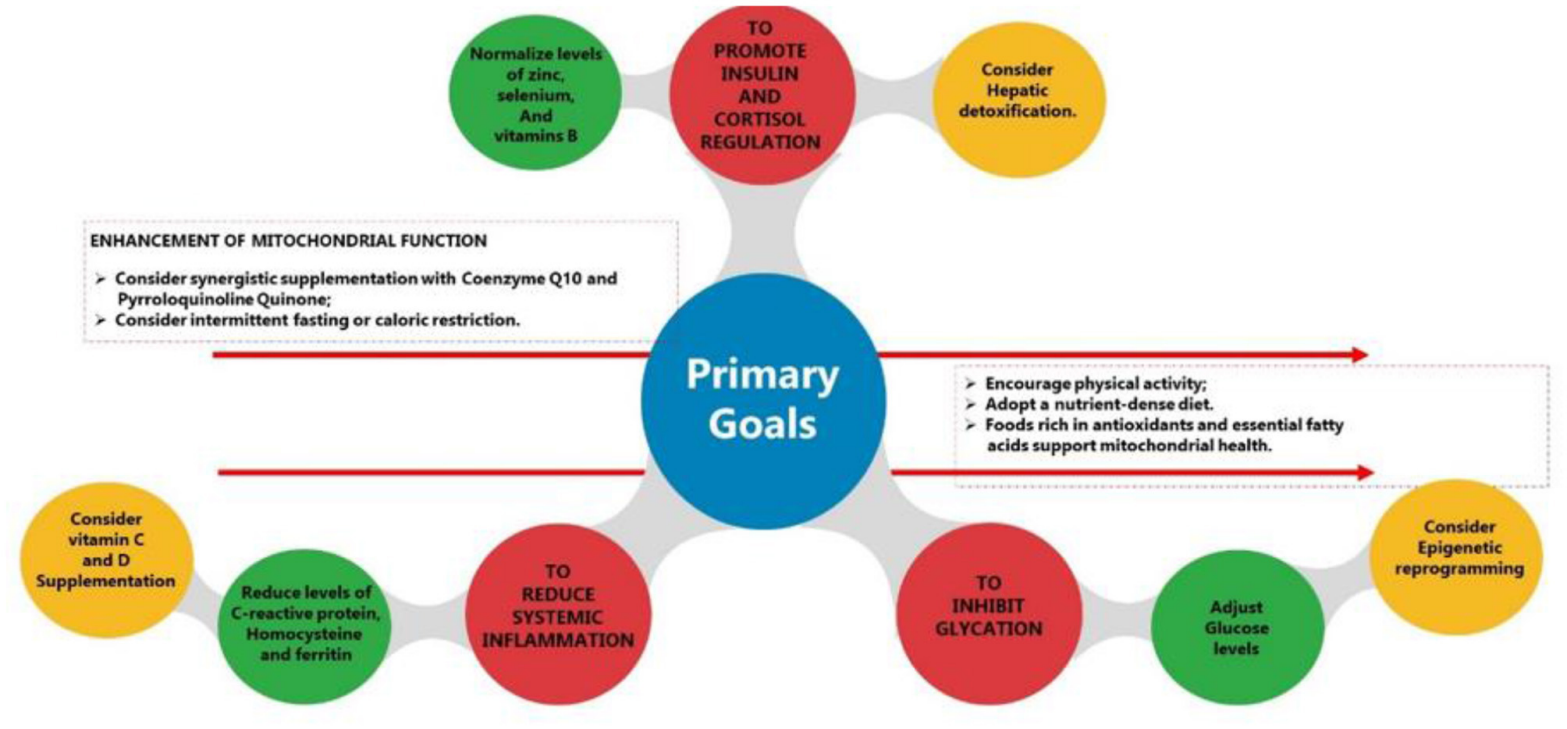
Biological drug delivery system primary goals.

To achieve these objectives, promoting synergistic metabolic interactions is crucial. Figure 6 presents an evidence-based model for achieving key therapeutic goals. Each component in this framework is supported by scientific literature and aligns with established physiological principles. The strategies outlined reflect a translational approach to optimizing metabolic health and drug delivery efficiency. Additionally, the concept of micronutrient synergy, examined in this study, suggests that specific nutrient combinations may enhance physiological responses, particularly in regulating insulin and cortisol levels, reducing inflammation, and inhibiting glycation (56–59). Table 12 details how micronutrient interactions contribute to these outcomes.

**Table 12:** Micronutrient synergism and the objectives of the clinical decision model.

**Objective**	**Description (56–59)**
Insulin and cortisol regulation	The synergistic effects of specific micronutrients enhance glucose metabolism and modulate the stress response, reducing insulin resistance and stabilizing cortisol levels. Magnesium and chromium improve insulin sensitivity, while vitamin C and pantothenic acid help regulate cortisol secretion, promoting metabolic balance and reducing susceptibility to endocrine disorders.
Inflammatory process modulation	Chronic inflammation is a key driver of various pathological conditions. Synergistic micronutrients such as omega-3 fatty acids, vitamin D, selenium, and antioxidants (vitamins C and E, zinc) exhibit strong anti-inflammatory properties. Their combined action strengthens the body's defense against oxidative stress and inflammatory cascades, creating a more stable internal environment conducive to drug therapy efficacy.
Glycation inhibition	Glycation leads to the formation of advanced glycation end-products (AGEs), which contribute to aging and chronic disease pathogenesis. Micronutrients such as vitamin B6, alpha-lipoic acid, carnosine, and benfotiamine act synergistically to reduce AGE formation, protecting protein structures and cellular integrity, thereby enhancing longevity and metabolic function.

The implementation of synergistic micronutrient strategies presents a holistic and scientifically grounded approach to chronic disease management. This conceptual framework acknowledges the complexity of human physiology and the need for multifaceted interventions to optimize therapeutic responses. As part of WP5, the hypothesis—proposing that a well-regulated physiological state, achieved through insulin and cortisol modulation, glycation inhibition, and inflammation control, can function as a biological DDS—was evaluated against SMART (40) and FINER criteria (41), demonstrating its specificity, feasibility, and clinical relevance (Table 13).

**Table 13:** WP5 criteria analysis.

**Criteria**	**Description analysis**
**SMART**	
Specific:	The hypothesis is specific as it outlines three precise clinical interventions: regulation of insulin and cortisol levels, inhibition of glycation, and reduction of inflammation. The goal is clear: to create a well-balanced bodily state that enhances drug delivery efficiency.
Measurable:	The hypothesis involves measurable outcomes, such as insulin and cortisol levels, markers of glycation (AGEs), and inflammatory markers. Success can be quantitatively assessed through improvements in these biomarkers and therapeutic outcomes.
Achievable:	The hypothesis is achievable with current scientific knowledge and clinical practices. There are existing therapies and interventions targeting each of the specified areas (hormonal regulation, anti-glycation, anti-inflammatory).
Relevant:	The hypothesis addresses a significant problem in medicine: the optimization of drug delivery and therapeutic efficacy. It aligns with the growing field of personalized medicine and its emphasis on tailored therapeutic approaches.
Time-bound:	The hypothesis implies a time-bound nature as clinical interventions would need to be monitored over specific periods to observe changes and improvements in biomarkers and therapeutic outcomes. Specific timelines for clinical trials and longitudinal studies could be established to evaluate the hypothesis.
**FINER**	
Feasible:	The hypothesis is feasible given current technologies and methodologies in endocrinology, pharmacology, and personalized medicine. Research and clinical trials can be designed to test the effects of the proposed interventions.
Interesting:	The hypothesis is intriguing as it suggests a novel approach to drug delivery, potentially transforming standard medical practices. It integrates multiple disciplines, which can attract a wide range of scientific and clinical interest.
Novel:	The concept of using a well-balanced bodily state as a biological drug delivery system is innovative. It combines traditional therapeutic goals with a new purpose, offering a fresh perspective on optimizing drug efficacy.
Ethical:	The interventions proposed (regulation of hormone levels, anti-glycation, and anti-inflammatory treatments) are ethically sound and align with standard clinical practices. The focus on minimizing side effects and maximizing benefits aligns with ethical principles of beneficence and non-maleficence.
Relevant:	The hypothesis addresses relevant health issues, such as diabetes, metabolic syndrome, and chronic inflammation. It contributes to the broader goal of improving patient outcomes through personalized and targeted medical approaches.

## 4 Discussion

The research process was meticulously structured using the Work Breakdown Structure (WBS) methodology, ensuring a systematic and step-by-step approach that provided detailed insights into each methodological component and its corresponding results. The integration of WBS facilitated the logical segmentation of research processes into interconnected phases, ensuring iterative refinement and cross-disciplinary integration (PMI, 2019; 2021) (20, 21). This methodological rigor was particularly advantageous for integrating diverse fields such as epigenetics, pharmacology, and drug delivery science, as it allowed for the structured incorporation of emerging insights into a coherent decision-making framework. In addition to structuring the study into modular Work Packages (WPs), WBS enabled the sequential identification of Fundamental Points of View (FPVs) and Critical Success Factors (CSFs), ensuring an optimal sequence for the application of Multi-Criteria Decision Analysis (MCDA). By organizing the decision-making process hierarchically, this approach enhances not only reproducibility but also the adaptability of systematic reviews, allowing for continuous refinement as new evidence emerges (35). Moreover, WBS promotes the seamless incorporation of emerging insights from epigenetics, pharmacology, and drug delivery science, while MCDA refines decision-making by categorizing critical evaluation factors into hierarchical components (23). The synergy between these methodologies has been successfully applied in translational healthcare models, particularly in decision-making frameworks that navigate complex biomedical scenarios (22, 60, 61).

The triangular clinical decision-making model developed in this study is based on the premise that the regulation of cortisol and insulin levels, inflammation reduction, and glycation inhibition collectively optimize drug absorption and systemic bioavailability. Cortisol and insulin modulation directly influence metabolic stress and transporter efficiency, as dysregulated levels contribute to altered membrane permeability and impaired intracellular transport (48, 49, 62–67). In particular, insulin homeostasis plays a pivotal role in glucose metabolism, which directly impacts the cellular uptake of pharmacological agents by modulating transporter expression and endocytotic pathways. By stabilizing glucose homeostasis and reducing catabolic effects, the model fosters a metabolic environment conducive to efficient molecular transport and cellular uptake, ultimately enhancing drug bioavailability and systemic distribution (62, 68). This metabolic fine-tuning not only improves drug absorption but also minimizes pharmacokinetic variability, ensuring a more predictable therapeutic response. Chronic inflammation, a key driver of endothelial dysfunction, restricts drug permeation by disrupting cellular communication (52, 69–76). Furthermore, glycation inhibition preserves membrane fluidity and zeta potential, reducing biochemical barriers that hinder receptor function and molecular transport (54, 77). The mitigation of advanced glycation end-products (AGEs) decreases oxidative stress, preventing modifications in protein transporters and increasing overall bioavailability of pharmacological agents (55, 78). Together, these three regulatory axes establish a physiological framework that optimizes pharmacokinetics while also mitigating long-term drug resistance, paving the way for more efficient and personalized therapeutic strategies. This synergistic interplay between metabolic, inflammatory, and glycation-related axes is not merely additive but interdependent, forming a dynamic physiological network in which modulation of one vertex reinforces the regulatory effects of the others, collectively enhancing membrane function, transport efficiency, and therapeutic responsiveness.

Crucially, this study conducted an exhaustive integrative analysis demonstrating that the regulation of cortisol and insulin, along with inflammation and glycation control, has the potential to mimic all Critical Success Factors (CSFs) observed in Drug Delivery Systems (DDS) activated by physical stimuli, as identified in Table 4. This mapping was meticulously detailed in Tables 8–11, reinforcing the conceptual validity of the triangular model as a Biological Drug Delivery System (BDDS). This translational approach thus represents a shift in drug delivery science, moving from a purely formulation-based model to a biologically integrated system where metabolic homeostasis itself becomes a determinant of drug absorption and efficacy (50, 63, 79). Unlike conventional DDS, which primarily focus on drug formulation and controlled release, this model emphasizes systemic optimization, preparing the biological environment to enhance drug absorption and therapeutic response.

By leveraging epigenetic modifications, micronutrient synergy, and improved nutrient bioavailability, the model aligns with the principles of translational and precision medicine, moving beyond conventional pharmaceutical modifications (3). Emerging insights into nutrient-epigenome interactions and their impact on drug transporter gene expression have begun to reshape our understanding of bioavailability modulation as an epigenetically regulated process. While classical mechanisms—such as DNA methylation, histone modifications, or non-coding RNA interactions—form the theoretical basis (3, 5, 6), their application here is not mechanistically dissected. Instead, these pathways were integrated into a decision-making model that emphasizes translational applicability. Due to the personalized nature of epigenetic programming and its inherent complexity, a detailed mechanistic mapping would require a level of description disproportionate to the study's scope. Therefore, this model incorporates epigenetic regulation as an operational mechanism—capable of modulating the physiological conditions needed for optimal drug delivery—rather than detailing the molecular steps involved in gene expression control.

This perspective repositions epigenetics from a passive background variable to a central pillar of the integrative therapeutic framework. It conceptualizes epigenetics as an active modulator of drug bioavailability by influencing membrane stability, intracellular signaling, and systemic regulation. As such, epigenetics reinforces the body's intrinsic capacity to optimize therapeutic outcomes and strengthens the foundation of a Biological Drug Delivery System that adapts dynamically to internal cues.

The flexibility of this model distinguishes it from rigid clinical protocols. Instead of defining fixed pharmacological regimens, it functions as a clinical decision-making framework, allowing for real-time adjustments based on patient-specific metabolic responses. This adaptability is particularly relevant in managing chronic diseases, where individualized interventions are crucial due to varying physiological profiles. For example, patients with metabolic syndrome may require different therapeutic adjustments compared to those with autoimmune disorders or psychiatric conditions, emphasizing the need for a tailored, dynamic strategy. The precision and safety of physiological stimuli-based DDS, such as heat therapy, ultrasound triggering, and electromagnetic modulation, depend on real-time biomarker monitoring, metabolic profiling, and continuous reassessment of patient responses. Incorporating AI-driven analytics into this framework could further enhance its clinical utility, enabling predictive modeling and real-time therapeutic adjustments based on patient-specific biomarkers. Ethical considerations are also paramount, ensuring patient autonomy, informed consent, and a thorough risk-benefit analysis in the clinical implementation of biological DDS strategies.

While this model represents an innovative approach to drug delivery, its clinical validation requires further refinement. Future research should focus on the development of AI-driven decision-support tools to assist clinicians in dynamically tailoring interventions, ensuring that physiological modulation strategies are seamlessly integrated into precision medicine rather than applied as generalized treatment algorithms. Additionally, biomarker-based patient stratification studies will be essential to evaluate the model's applicability across different populations, refining the decision-making framework for specific metabolic, inflammatory, and epigenetic profiles. These next steps will be critical in transforming this conceptual model into a clinically validated therapeutic strategy. The effectiveness of this model relies on further research and clinical validation, particularly regarding the previously identified personalized stabilization period, estimated between 10 to 20 weeks. By introducing the concept of a biological drug delivery system, this study expands the conventional understanding of drug delivery by incorporating human physiological responses as an integral component of therapeutic optimization. Rather than solely focusing on pharmaceutical modifications, the model emphasizes the importance of balancing nutrient availability, hormonal regulation, and cell membrane integrity to enhance medication efficacy.

## 5 Conclusions

This research introduces a novel therapeutic intervention based on two systematic literature reviews, focusing on the role of physical stimuli in enhancing drug delivery. By employing the open innovation method, the triangular clinical decision-making model presents a promising framework aligned with the principles of personalized medicine and nutraceuticals. The model acknowledges the significant influence of epigenetics and nutrition on medication response, integrating these factors into a structured approach aimed at improving drug absorption, metabolism, and targeted therapies. This integrative perspective has the potential to advance treatment efficacy by optimizing biological receptivity to pharmacological interventions.

The effectiveness of this model relies on further research and clinical validation, particularly regarding the previously identified need for a personalized pre-treatment stabilization period, aimed at optimizing metabolic and physiological balance prior to pharmacological interventions. Moreover, the continuity of this physiological modulation during treatment may prove essential to maintain therapeutic responsiveness and minimize pharmacokinetic variability. By introducing the concept of a biological drug delivery system, this study expands the conventional understanding of drug delivery by incorporating human physiological responses as an integral component of therapeutic optimization. Rather than solely focusing on pharmaceutical modifications, the model emphasizes the importance of balancing nutrient availability, hormonal regulation, and cell membrane integrity to enhance medication efficacy.

Although this study presents a theoretical model, its clinical implementation requires additional empirical validation. The proposed framework aligns with precision medicine by emphasizing physiological optimization as a strategy to enhance drug bioavailability, rather than relying exclusively on DDS formulation-based approaches. In this context, biomarker-driven assessments will be essential for defining personalized therapeutic strategies, reinforcing the translational potential of this model. Future research should focus on establishing structured clinical protocols, ensuring that proposed interventions can be tailored to patient-specific needs while maintaining therapeutic efficacy.

The theoretical-methodological framework proposed in this research serves as a reference for future translational studies, encouraging the integration of complementary approaches and reinforcing the value of orthomolecular medical practices. Additionally, the model highlights the critical role of personalized therapeutic strategies in clinical settings, ensuring that interventions are adapted to individual physiological needs. Future investigations should further explore and refine this approach to enhance body homeostasis and therapeutic responses, ultimately validating its efficacy and expanding its application across diverse healthcare contexts.

## Data Availability

The original contributions presented in the study are included in the article further inquiries can be directed to the corresponding author/s.
